# *PolarEyes* with enhanced digital imaging, a high-throughput solution for locating and grading crystals

**DOI:** 10.1107/S1600576726007727

**Published:** 2026-09-25

**Authors:** Thomas D. Smith, Alexandra Longcake, Michael J. Hall, Michael R. Probert

**Affiliations:** a School of Natural and Environmental Sciences, Newcastle University, Newcastle upon Tyne NE1 7RU, UK; b Indicatrix Crystallography Ltd, Newcastle upon Tyne, UK; University of Utah, USA

**Keywords:** crystallization, high-throughput experiments, automation, robotics, software

## Abstract

The long-range internal order of crystals enables them to exhibit a well known and long-exploited phenomenon: birefringence. Simple assessment of the interaction of crystals with a polarized light source has guided the hand of many crystallographers through history in matters of crystal selection. Historically the interaction of plane-polarized light has been analysed manually by the user. However, adapting the use of a motorized polarized light microscope to a modern small-molecule-optimized high-throughput workflow can allow for more efficient screening of crystals, in turn alleviating the analysis bottleneck previously encountered in parallel crystallization techniques, such as encapsulated nanodroplet crystallization. Presented herein are developments to an existing open-source imaging robot, including the novel integration of a commercial imaging device and a new software suite for analysis. The open-source program *PolarEyes* is presented for the extraction of polarization quantities from optical light micrographs. Included in *PolarEyes* are facilities to perform normalization against a background image and false-colour visualization using the hue, saturation, luminance colour model, and capabilities to perform analysis of multiple different incident polarization states.

## Abstract 2

An open-source program for the calculation and visualization of polarization-derived properties of crystalline material is described, alongside the integration of a polarizing image sensor into a home-built high-throughput imaging setup.

## 1. Introduction

Small-molecule crystallization has historically been considered by many (both within and outside the crystallographic community) to be a ‘dark art’. This reputation is not entirely undeserved: the crystallization behaviour of structurally similar species can appear to vary wildly, and even chemically simple molecules can provide challenges to success. Despite this, and with single-crystal X-ray diffraction (SCXRD) considered the ‘gold standard’ of analytical techniques, crystallization of small molecules is an essential step in the pipelines of many chemical industries, including the development of novel pharmaceutical agents.

Pharmaceutical development, especially in the early and exploratory phases of compound identification, can encounter difficulties in producing single crystals that can be generalized into two broad categories: limited sample quantity as a result of small-scale or low-yielding synthesis; and the ever-increasing chemical complexity and diversity of drug candidates. Traditional crystallization methods, such as those outlined by Jones (1981 ▸), are often inappropriate in this context, typically requiring many milligrams of material to explore the crystallization behaviour of the test species in a single condition. Other compounding factors, such as high degrees of conformational freedom, can further lower the chance of crystallization success (Wicker & Cooper, 2015 ▸), with knock-on effects to the overall throughput of a drug discovery pipeline.

The problematic combination of limited sample availability and large species that are difficult to crystallize is well known among the structural biology community, who routinely perform crystallographic analysis of structures where a ‘high degree of conformational freedom’ is something of an understatement. The generation of crystals suitable for SCXRD in this field has been assisted by performing large numbers of small-scale crystallization experiments in parallel, studying different combinations of conditions in each experiment. These experimental design schemes were pioneered as early as the 1970s with incomplete factorial experiments (Carter, 1979 ▸) and successive automated grid searches (Cox & Weber, 1988 ▸) that have since become commonplace in the modern structural biology laboratory.

While pioneered and honed by structural biology, techniques such as microbatch-under-oil (Chayen *et al.*, 1992 ▸; Babor *et al.*, 2019 ▸) and encapsulated nanodroplet crystallization (ENaCt) (Tyler *et al.*, 2020 ▸) show that parallel methodologies are also applicable to a range of small molecules. However, while the methodologies are relatively easy to adapt in theory, wider-scale adoption and overall throughput are being held back by a lack of specialized resources. While some hardware from protein workflows may be directly ported into small-molecule pipelines (the ENaCt technique uses an SPT Mosquito Xtal3 with no modification, to great success) to enable small-molecule crystallizations of chemical species on a 96-well plate, in other areas problems may be encountered.

Experimental analysis, for example, is a time-consuming task if performed manually on a traditional microscope: every well of every multi-well crystallization plate must be optically analysed to check for the presence or absence of crystals. A solution would be for a department to purchase, or otherwise make use of, a ‘plate reader’ (sometimes also integrated into a ‘plate hotel’). These are often large and expensive devices which combine condition-controlled high-volume plate storage with automated microscopy (very often including fluorescence), a sample-tracking system and experiment design software, to provide a tightly integrated experience for the user.

Despite their unquestionable utility, many of these plate-reading devices are not optimized for use in small-molecule contexts. Their high cost and large footprint can render them financially or logistically unfeasible for many smaller laboratory settings, and their microscopy capabilities are simultaneously over- and under-equipped: many small molecules are not fluorescent in the visible range and the process of adding fluorescent tags is rarely beneficial; and polarization – a mainstay on traditional microscopes used for small-molecule work – is noticeably absent. Even within the structural biology community, the inconveniences of commercial designs are an acknowledged problem, and work such as that published by Bohm (2018 ▸) shows that the ‘minimum viable product’ for a plate reader can be constructed inexpensively using off-the-shelf components.

Bohm’s design is based around a ‘hobby-grade’ desktop engraving machine; the use of that descriptor is not to disparage the design, and in fact is a indicator of the variety and quality of hardware available to the ‘do-it-yourself’ scientist. The system presented by Bohm (2018 ▸) incorporated a stand­alone lens and microscope camera to capture images from multi-well crystallization plates, but it was also shown that the same translation hardware could be used unaltered with a traditional stereomicroscope. In the standalone setup, a 1 W white LED was used to illuminate the wells at a grazing angle to highlight artefacts better. We were interested in this design and hypothesized that, by optimizing the light source to include polarization, it could demonstrate the potential to alleviate the analysis bottleneck in the ENaCt-based high-throughput small-molecule crystallization pipeline.

It has long been known that non-isotropic materials will rotate the plane of plane-polarized light incident upon them, and this remains a simple yet powerful method of crystal identification and quality assessment. Despite small molecules often crystallizing in non-isotropic systems [∼25% of entries in the Protein Data Bank (PDB, https://www.rcsb.org/; Berman *et al.*, 2000 ▸) are in the space group 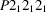], the prevalence of vacuum-formed or extruded plastic multi-well microplates in biological settings has resulted in polarized light being under-utilized in protein crystallization equipment. This is not to say that all small molecules crystallize in anisotropic systems, but the prevalence of such crystal systems (particularly triclinic and monoclinic) in the Cambridge Structural Database (CSD; Groom *et al.*, 2016 ▸) highlights how generally applicable the technique remains.

Table 1 ▸ shows the distribution of crystal systems in the CSD (as of the 2026.1 release, June 2026) generated using the CSD Python API (Version 3.4; Sykes *et al.*, 2024 ▸). It shows that, for entries fitting our criteria (organic, not polymeric or organometallic, and having 3D coordinates), ∼98.3% of those structures are reported in space groups belonging to potentially anisotropic crystal systems. A further ∼1.5% of structures are only isotropic in one direction and so, depending on growth orientation, would still be revealed using polarized light.

A similar analysis was performed using data obtained from the PDB using the *rcsb-api* Python toolkit (Piehl *et al.*, 2025 ▸). This showed that ∼90% of the 205859 structures checked are in potentially anisotropic crystal systems, highlighting that polarization remains an invaluable tool for analysing crystals in a very general sense. This conclusion is reinforced by surveying the Inorganic Crystal Structure Database (ICSD; Zagorac *et al.*, 2019 ▸). Although it contains a higher prevalence of entries in higher-symmetry systems, polarized light would find application in almost 80% of reported structures there.

The use of ‘potentially’ as a qualifier for anisotropic in the preceding discussion is in reference to the probability that the view direction through a crystal is anisotropic. That is to say, the cross section of the optical indicatrix along the light beam is circular, and both the ordinary and extraordinary rays will experience different refractive indices. For cubic systems, the optical indicatrix is spherical and therefore there is no view direction along which the material is birefringent. For all other systems, there is only one such view direction (hexagonal and tetragonal, known as *unixial*) aligned with the crystallographic axes, or two (orthorhombic, monoclinic and triclinic, known as *biaxial*) that are not necessarily aligned with crystallographic axes.

In this work, we show that an automated device can be constructed to take full advantage of the interaction between a crystalline material and polarized light, while only using off-the-shelf hardware and open-source software. We then demonstrate its implementation as part of a high-throughput small-molecule crystallization regime through the crystallization of a test species, benzo-2,1,3-thiadiazole-4-sulfonyl chloride (**A**).

## 2. Polarization imaging and automation

Integrating polarization imaging into an automated system is not without challenges. When using a standard optical microscope, a fixed linear polarizer is installed above the light source, and another rotatable one between the sample and the eyepieces (the *source* and *analyser* polarizer, respectively).

A possibly crystalline sample is placed on the stage and the analyser polarizer is rotated such that it is 90° offset from the source: the polarizers are ‘crossed’. This arrangement should allow no light to reach the eyepieces unless it has passed through a birefringent material, producing a black background against which a sample will appear to ‘shine’. Rotating the analyser polarizer slightly around this crossed position can give an impression of how consistent the effect is, although it is difficult to obtain any quantitative information from this process. It is of course also possible to rotate the sample instead of the analyser polarizer to achieve a similar effect. This is shown schematically in Fig. 1 ▸ (left), wherein the crystal is shown as a purple block on the lower branch of the diagram. After passing through the crystal, the angle of the linearly polarized light has been rotated, allowing it to pass through the second ‘crossed’ polarizer. Rotation of the analyser (or the crystal) will result in the contrast curve shown in Fig. 1 ▸ (right).

The simplest integration solution, wherein a second polarizer is rigidly mounted with a 90° rotational offset to the first (*i.e.* in a crossed arrangement), has an incredibly low cost/benefit ratio: it requires no additional software or hardware, while mostly mirroring the traditional method. In this configuration an automated instrument would collect a single image per crystallization well, wherein black pixels correspond to regions of the well in which either no material has grown or the material that has grown is isotropic along the view axis, and non-black pixels correspond to regions containing non-isotropic material. From an image processing and computer vision perspective, this solution is incredibly inviting: it produces an image where only non-black pixels are interesting, skipping many steps in a traditional computer vision pipeline up to and including binarization. If the assumption can safely be made that any non-isotropic material is a single crystal of the analyte, then a simple crystal detecting system could be built from this.

Despite its appeal, this setup does not actually provide any characterization of the light incident on the camera sensor, and so deriving quantitative information about the interaction between light and a crystalline material is impossible.

Linearly polarized light can be considered a special case of electromagnetic (EM) radiation, and as such can be described by the Stokes parameters (Stokes, 1851 ▸). A full definition of the parameters is deferred until Section 3, but in brief, in order to give a full quantification of the polarization state of EM radiation it is necessary to measure intensities with an analyser polarizer set to four different, evenly spaced, angles. This rules out the single-angle solution, and would seem to imply the need to have a mechanism allowing the analyser polarizer to be automatically rotated.

We were keen to avoid using additional components in our system, both to keep in line with the low-cost philosophy of the design we were modifying and to minimize the cost and complexity of the final product. More components introduce more points of failure and, in an instrument designed to run as part of a high-throughput system, failures and downtime are counterproductive. Similarly, requiring more motion to be performed as part of the instrument’s run procedure causes a reduction in throughput, as extra dwell time is required for motors to arrive at their desired position and for residual vibrations to dampen. Finally, it is possible that the rotation resulting from a motorized component may not be exactly 45°, introducing small errors to the calculation which are not necessarily trivial to correct.

A similar problem has been encountered by Kaminsky *et al.* (2007 ▸) in their construction of a birefringence-based imaging system. Their solution involved using an optical multiplexer to place four separate linear polarizers at 45° to one another before the camera sensor, thus dividing that sensor into four quadrants. This is a very attractive solution given that it requires no additional motorized or electronic components, but the specific implementation has downsides which we deemed unacceptable. Firstly, the linear polarizers must be manually aligned and calibrated to ensure the rotation is correct, and this is a time-consuming process which may need repeating periodically, introducing an extra maintenance burden. Secondly, the optical multiplexer used involves beam-splitting prisms and extra polarizing filters, all of which add monetary cost to the build and lead to increased light loss, requiring a higher incident illumination – not to mention that the multiplexer itself is not inexpensive. Packaging those extra components can also become a challenge in the pursuit of a compact easy-to-use setup.

The solution we chose for our system was instead to utilize a camera sensor to which a four-directional polarization grid has already been bonded. Sony released such a sensor in 2018, aimed at the industrial machine vision market who also use the polarization state of incident light to perform object detection, as well as to remove reflections when imaging through reflective transparent surfaces.

As opposed to the approach of Kaminsky *et al.* (2007 ▸), where the same image is passed through four filters and imaged simultaneously, the Polarsens sensors arrange the polarizers much more akin to the layout of a Bayer grid with repeating four-pixel (2 × 2) blocks (shown schematically in Fig. 2 ▸). The Bayer grid design, although undoubtedly an engineering and manufacturing triumph, has flaws intrinsic to its design: spatial distortion and the moiré effect.

The 2 × 2 block arrangement of angles allows for the Stokes parameters to be calculated for each block, enabling the creation of new images for each quantity with pixel dimensions half those of the sensor in each direction. This process immediately raises an issue with the grid nature of the polarizers: each of the polarization angles is sampled at a different point in space, meaning that there will be some distortion caused by the spatially separated intensities being collapsed into a single value. In practice, we find the effects of this to be unnoticeable, most probably due to the small size of the distortions as a result of the small real-space size of each pixel after magnification.

This camera sensor allows for a completely solid-state single-component solution to obtaining the maximum amount of information about the polarization state of light that has passed through a sample. Note that the Kaminsky *et al.* (2007 ▸) design for birefringence imaging could also benefit from this approach, and this is something we have trialled with our system to great success. As such, we developed the *PolarEyes* software, described below, to be fully compatible with both linearly and circularly polarized incident light.

## 3. The Stokes parameters

Earlier it was mentioned that the interaction between polarized light and a crystal could be quantitatively characterized using the Stokes parameters 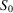, 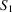, 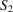 and 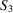 (sometimes referred to as *I*, *Q*, *U* and *V*, respectively). 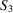 refers to the handedness of the polarization ellipse, and for linearly polarized light this can be discarded. As such it will not be discussed further.


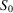
 represents the total light intensity falling on a pixel, and is usually calculated using measurements taken without the polarizer in place. However this is not possible with the analyser polarizer being bonded to the camera sensor, so instead we calculate 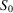 according to 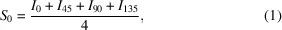
where 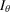 corresponds to the intensity of the pixel under the polarizer at θ degrees.


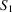
 and 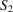 are obtained trivially: 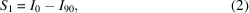

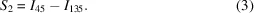
These two values can be combined to calculate what is formally referred to as the inclination of the polarization ellipse, although for our purposes it can be thought of as the angle of the plane-polarized light relative to the 0° polarizer on the camera. The calculation of this is shown in equation (4),
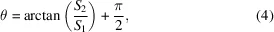
with the addition of 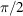 to shift the arctan function’s output to be entirely positive, purely for programming convenience. The exact real-space direction of this angle is unknown, and for our purposes is unimportant. Finally, a further quantity known as the ‘degree of polarization’ (DoP) may be obtained, which describes the ratio of the polarized intensity to the total incident intensity. This may be obtained from 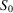, 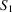 and 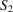 by equation (5),
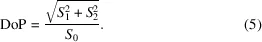
Generally, the DoP would also take into account the handed­ness of elliptically polarized light, but as mentioned earlier we are focusing only on linear polarization and so the contribution of 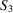 is ignored.

## 4. The *XtalEyes* imaging system

As part of our work with the ENaCt technique, we have developed a software/hardware ecosystem to improve both experimental efficiency and data reliability. This ecosystem, named *Atomicity*, is built around two core concepts: a central repository of experimental data and a low-cost automated plate-reading device (shown in Fig. 3 ▸). A full exploration of the former is out of the scope of this work, but insofar as it is relevant it will be discussed here.

Our plate-reading device, as previously mentioned, is based on the design of Bohm (2018 ▸) with modifications made to integrate a polarized light source. Integrated also was a commercially available implementation of the aforementioned Sony IMX250MZR Polarsens sensor: the Jai Go5100MP-USB. No major modification was made to the mechanical design, only the addition of a 3D-printed plate holder, the better to accommodate the 96-well Swissci Laminex LCP plates used.

A new light source was constructed using a high-power white chip-on-board (COB) light-emitting diode (LED, Cree CXB3070) driven in a constant-current configuration at 350 mA. A COB LED was chosen for the balance of low cost, high brightness and ease of packaging that they offer. It is only necessary to provide them with sufficient cooling to ensure that the junction temperature of the individual diodes does not rise above the datasheet-specified maximum. Our implementation drives the LED far below its rated maximum and so the heat output is minimal: a heat sink placed in the path of a 50 mm 12 V fan is more than sufficient to prevent temperatures rising too high, even with long periods of operation. High temperatures around the light source would also cause problems with our use of polylactic acid (PLA) filament for the majority of our printed parts, some of which are in either direct contact or very close proximity to the COB itself. PLA will start to soften and lose structural integrity above 60°C, and so in addition to cooling the LED’s heat sink, some of the fan’s output was directed past the most vulnerable components. The use of PLA in our design was a prototyping convenience – depending on the final application and operating environment of the instrument, any number of materials could be substituted instead, removing any issues regarding heat tolerance.

Other light source options were considered: monochromatic LED arrays, commercially available fibre light sources or even a traditional halogen bulb. However, all other options were deemed unsuitable on the grounds of high cost, undesirable spectral output or packaging difficulties.

Microscopy of any flavour benefits greatly from a bright and even illumination field, and while we found that the COB module has satisfactorily homogenous intensity across the camera’s field of view, a ground-glass diffuser (Thorlabs, DG10-1500) was placed in the beam path before the polarizer cassettes to reduce any inhomogeneity that remained. Additional corrections are performed by *PolarEyes* during software processing (see Appendix *B*[App appb] for details). Bright illumination is preferable for automated imaging because faster shutter speeds and lower gain values are feasible, reducing the chance of motion blur and reducing noise, respectively. To this end we chose the LED drive current, which is related to the light output of the LED, such that after the light has passed through all filters the camera’s shutter speed was just higher than the supported minimum (100 µs in our case). Due to the inverse relationship between image brightness and magnification, it was also necessary to ensure that the source had sufficient light output to allow for low gain and shutter speed values in the future if higher magnification optics were to be employed.

In addition to the alterations made to the light source itself, we designed a mounting system that allows different combinations of polarizing filters to be inserted into the light path before it is incident on the sample. Up to two filter optics, each mounted in ‘cassettes’, may be inserted. The cassettes themselves are sized to hold optics up to 50 mm in diameter to give the most flexibility when purchasing polarizers, which must have diameters at least as large as the field of view of our C-mount camera. Some cassettes incorporate the ability to rotate the filter using a worm drive arrangement (shown on the right-hand side in Fig. 4 ▸) for situations where the relative rotation of polarizers is important (when generating circularly polarized light using a linear polarizer and quarter waveplate, for example). The T-shaped rail holding the cassettes to the mounting system is a close running fit to allow for cassettes to be inserted and removed as required without compromising on repeatability.

The exact software model for the plate reader is not materially relevant to the discussion at hand. In brief, the instrument’s physical configuration (installed filters, zoom level *etc.*) is read in from a configuration file when the control software starts, and these metadata are propagated through to any data captured by the system.

Before the start of an imaging run, an output root directory is created (the path of which is derived automatically from metadata about the selected experiment) in which a set of subdirectories are generated and special files written. This run output structure is standardized to allow other programs (both internal and external to the *Atomicity* ecosystem) to interact with produced data.

After successfully building the output structure and ensuring the hardware is initialized, control is passed to a user-provided Lua script. Each script may provide setup and tear-down code, providing the ability to initialize any hardware/image processing routines and then safely de-initialize resources afterwards. Errors are propagated back to C++ code and handled as appropriate.

By the end of an imaging run, an image from each visited well (as well as a background image taken with the plate clear of the camera) is saved to disk as an HDF5 file (https://www.hdfgroup.org/), within which each of the sensor’s four polarization angles will be stored in a separate dataset. Optionally, an HTML report may also be generated, containing experimental metadata alongside captured images after processing and conversion to portable network graphics (PNG) format.

*XtalEyes* does not perform any polarized light post-processing: it serves only to provide an interface to the experiment database and to control the plate-reader hardware. What little processing it performs is either to correct errors introduced by the hardware, to change the shape of captured data or to ensure that as much of the metadata as possible is associated with the data. Instead, a separate program to perform more extensive processing was written using the Rust programming language (https://www.rust-lang.org), namely *PolarEyes*.

## 5. PolarEyes

*PolarEyes* was, by design, kept separate from the main software suite primarily for ease of distribution – it has utility outside of the *Atomicity* ecosystem and therefore does not incorporate any specific ideas related to the wider experiment data model. Instead, it reads a *.h5 file from a specified directory, performs the processing algorithm described, and saves the resulting outputs either as false-colour images or directly as new HDF5 files, with each of the calculated quantities saved as a separate dataset.

A companion program included as part of the main *PolarEyes* distribution, *amygdala*, simplifies the process of running the *PolarEyes* algorithm for the outputs of an *XtalEyes* imaging run. Instead of processing each output image serially, the *Rayon* library (https://github.com/rayon-rs/rayon, Version 1.10.0) is used to perform all processing in parallel, automatically scaling to the number of CPU cores available.

At its heart, the *PolarEyes* application is an optimized implementation of equations (1) to (5). Extensive use is made of the *ndarray* library (https://github.com/rust-ndarray/ndarray, Version 0.16.1) and its parallel iterator support to perform calculations on large input pixel arrays in reasonable time frames. The aim of *PolarEyes* was *not* to be a real-time implementation of this algorithm but instead to be a component of an offline processing pipeline. This relaxes the performance constraints significantly, although care was still taken to ensure that the performance was not excessively slow such as to preclude its use in a soft real-time environment if required.

A block diagram of the system is shown in Fig. 5 ▸.

### 5.1. Visualization

False colouring is a common practice for pseudo-image data, particularly where the absolute value is not the most important takeaway from a visualization. Given a primary aim of the system is to provide rapid imaging of multi-well plates and identify regions of crystallinity, false colouring was a natural choice. When designing any data visualization scheme, it is important to consider the ‘learning curve’ attached to it, *i.e.* how easily a user can be trained to interpret and analyse correctly the visualization presented to them. For this application, the exact colour produced is not materially relevant, reducing the interpretation process down to finding blocks of similar colour and then assessing their shape (a skill with which all crystallographers are well versed).

With false colouring being so ubiquitous, there are many approaches one could take to convert a scalar value into a coloured pixel. In our opinion, the RGB colour model (the one with which most people are already familiar) does not lend itself well to this purpose: the three-component nature of an individual colour leads to interesting challenges regarding how a single scalar value should be mapped to the RGB tuple. Interpretation is also challenging as it can lead to users needing to determine if a region is ‘more blue’ or ‘less green’ than another, something which is not necessarily feasible on some displays even if it were intuitive.

Instead, inspired partly by the fact that the scalar quantity requiring conversion already represents an angle, we chose to use the HSL (hue, saturation, luminance) colour model. In this model, hue is represented as an angle between 0° and 360°, and our polarization angles are stored in the range 0° to 180°, making the mapping incredibly intuitive. Luminance and saturation are set to constant values half-way through their respective ranges for maximum legibility. When considering the ‘legibility’ of the visualization we were quite satisfied; however, an improvement one could make would be to change to a perceptually uniform colour space such as OKLCh (the polar form of the popular OKLab), which shares the ‘hue as an angle’ rationale.

All quantities are able to be false-coloured, and this is performed using the same strategy of remapping the natural ranges of the quantity onto the range of hue.

### 5.2. Interpretation

At present, the system does not provide an automated interpretation routine. Instead, it is left to individual users to assess whether a crystallization well may contain material of interest, using the false-coloured outputs from *PolarEyes* as a guide. Specific examples of outputs are discussed later, but in brief single crystals will generally appear as blocks of solid colour in both the extinction angle and linear DoP images. The specific colour is of little interest; instead it is the *consistency* that the user is assessing.

Users will typically copy the *PolarEyes* outputs to their own workstation (along with the HTML report discussed later) and perform an initial pass wherein they shortlist candidate crystals for further analysis. A well is then inspected under a traditional binocular microscope to confirm or disprove initial suspicions, before crystals are extracted from the plate by removing a small section of the glass cover slip with a carbide scriber. This pre-screening step represents an improvement both in user comfort and in efficiency. Although analysis on a traditional microscope is still involved, checking only a small number rather than the full 96 wells represents a potential increase in throughput.

## 6. Representative system performance

In order to demonstrate the system in action, an ENaCt experiment was performed on a small pharmaceutically relevant compound. This is a representative example of how the system is used in our laboratory day to day. 2,1,3-Benzothiadiazole-4-sulfonyl chloride (**A**) is a common precursor to sulfonamide-containing medicines.

After preparation, the crystallization plate was imaged approximately every 24 h for four days and twice more on the sixth and seventh days, producing 576 images totalling 2.9 GB of raw binary data. During the HTML report generation introduced in Section 4, each polarization angle is converted into an image in the PNG format and then merged with the four false-coloured image outputs from *PolarEyes*. This produces a further ∼400 MB of image data used for generating user-facing HTML reports. An example of this eight-component collage is shown in Fig. 6 ▸, with each panel labelled in the top right. Scale bars are automatically derived using the instrument’s installed magnifying optics, with the plate code and well address pulled from the embedded metadata. The colour of image annotations (scale bars, plate codes *etc.*) is automatically determined on the basis of the average colour of that region of the image (hence the change from white text to black text as the background gets lighter).

Each imaging run on our home instrument takes ∼5 min, plus a further 2 min for post-processing. This post-processing speed is primarily limited by the read/write times of the network-attached storage unit that is used as our bulk data store, as collage creation is very read intensive.

The produced images are then used to inform (either in whole or in part) the ranking assigned to a given well on a crystallization plate. The ENaCt technique uses five categories to discretize the continuum of possible crystallization outcomes into a tractable and useful system, and representative examples of four of these categories are shown in Fig. 7 ▸. Not shown is an example of the ‘F’ category, as this is a robotic failure resulting in no dispensed liquids. In the sample experiment, none were observed. These images are the greyscale intensities of the 135° polarizer, as in our setup that happens to be the ‘uncrossed’ angle.

While it may be possible to spot regions of interest from these greyscale images alone, it is more illustrative to look at the outputs of *PolarEyes* instead, and these are shown in Fig. 8 ▸. Fig. 8 ▸(*a*) shows no crystalline material of any kind and is representative of an experiment which is still ongoing. This particular image was taken immediately after the plate was made, whereas all others in Figs. 7 ▸ and 8 ▸ were taken after seven days. The combination of acetonitrile and Fomblin-Y has yet to produce any kind of result and so will be left alone and rechecked on subsequent imaging runs. Fig. 7 ▸(*b*) shows a crystallization which has ‘crashed out’, which is to say that the substrate left the solvent too quickly and was not able to form single crystals fully. Fig. 8 ▸(*b*) does show that the solid is crystalline, but it is likely to be a crystalline powder and therefore unsuitable for SCXRD.

Fig. 7 ▸(*c*) shows very small single crystals which, although not suitable for in-house diffraction equipment, may provide structural data. The greyscale image shows small and needle-like crystallites, one of which may yield usable data at a central facility, but taking a crystal from this well should be considered a last resort due to their low quality. The corresponding extinction angle plot [Fig. 8 ▸(*c*)] confirms that the material is probably crystalline, but the small size precludes a simple structure solution.

Fig. 7 ▸(*d*) shows a single crystal which would most likely yield satisfactory structural information when analysed using an in-house diffractometer. In fact, this crystal was analysed using our in-house Rigaku XtaLAB Synergy-S diffractometer, producing a fully complete dataset in under just under 15 min. This crystal was chosen due to a combination of its large size (∼700 × 40 µm before cutting and mounting) and the consistency of the polarization angle across the majority of the surface [as shown in Fig. 8 ▸(*d*)]. The small non-uniform area at the left-hand end was a collection of small crystals which had grown over the main crystal, and these were removed prior to mounting.

The structure refined from the selected crystal is shown in Fig. 9 ▸, and the low values of both *R*_1_ and *R*_int_ show that the selection rationale is valid.

Upon repeating the same experimental conditions as described in Appendix *A*[App appa], a second polymorph of **A** was discovered. Conformationally the two polymorphs are almost identical, which is unsurprising given the lack of conformational flexibility, but instead they differ in their 3D packing. The ability to explore the solid-state landscape of small organic molecules is something for which ENaCt is gaining attention (Weatherston *et al.*, 2025 ▸).

As part of the repeated experiment described above, a time-lapse study was performed using the dedicated function provided by the *XtalEyes* software and microscope. The plate was mounted on the microscope for 24 h, during which an imaging cycle was automatically performed every 20 min. This imaging frequency was chosen as a compromise between temporal resolution and data volume. In total, 6912 images were captured over this period with an accompanying storage footprint of ∼35 GB.

The *ImageMagick* (https://imagemagick.org/) tool was used, in concert with some custom preprocessing to create the individual frames, to produce animated GIFs of each well on the 96-well crystallization plate. These were studied to investigate the crystal growth behaviour of each condition and could be used to correlate growth times or ‘time to first nucleation’ with crystallization conditions. Selected frames from the growth of a crystal of **A** (polymorph I) are shown in Fig. 10 ▸. These show the steady growth of a needle from the top of a droplet (*i.e.* the droplet–air interface) over the first 3 h of the experiment.

Recording a time-lapse video with a level of consistency in both time and location without the aid of an automated instrument is unfeasible in most scenarios, and although a single time-lapse study would occupy a single microscope for the duration, the low cost and simplicity of the presented design would allow for a laboratory to construct multiple versions if so desired.

## 7. Conclusions

Presented in this work is a low-cost plate reader that takes full advantage of polarized light to improve the throughput of the identification of crystalline material in small-molecule crystallization workflows. The plate reader has been in service in our laboratory for 18 months and in that time has performed nearly 1400 imaging runs, totalling over 130000 images and over 550 GB as of March 2026.

We have also presented an open-source software project, *PolarEyes*, which can extract valuable information from datasets consisting of four images captured with an analyser polarizer rotated by 45° increments.

Finally, we have presented a new application for an existing piece of hardware – the Sony PolarSens series of sensors and the JAI Go5100MP implementation – as an integral part of an automated small-molecule-oriented polarization microscope.

## Supplementary Material

Crystal structure: contains datablock(s) 2025ncs0268_p4a8, tds2026001_1_a6_fa. DOI: 10.1107/S1600576726007727/har5006sup1.cif

Structure factors: contains datablock(s) 2025ncs0268_p4a8. DOI: 10.1107/S1600576726007727/har50062025ncs0268_p4a8sup2.hkl

Structure factors: contains datablock(s) tds2026001_1_a6_fa. DOI: 10.1107/S1600576726007727/har5006tds2026001_1_a6_fasup3.hkl

CCDC references: 2469226, 2536919

## Figures and Tables

**Figure 1:**
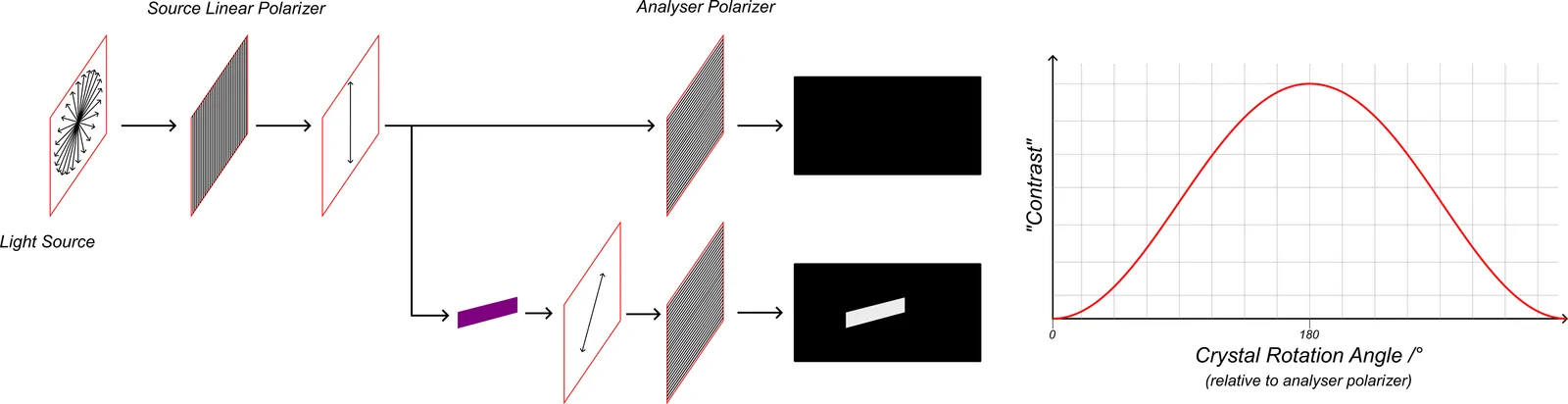
Schematic representation of the traditional polarization imaging of crystals.

**Figure 2:**
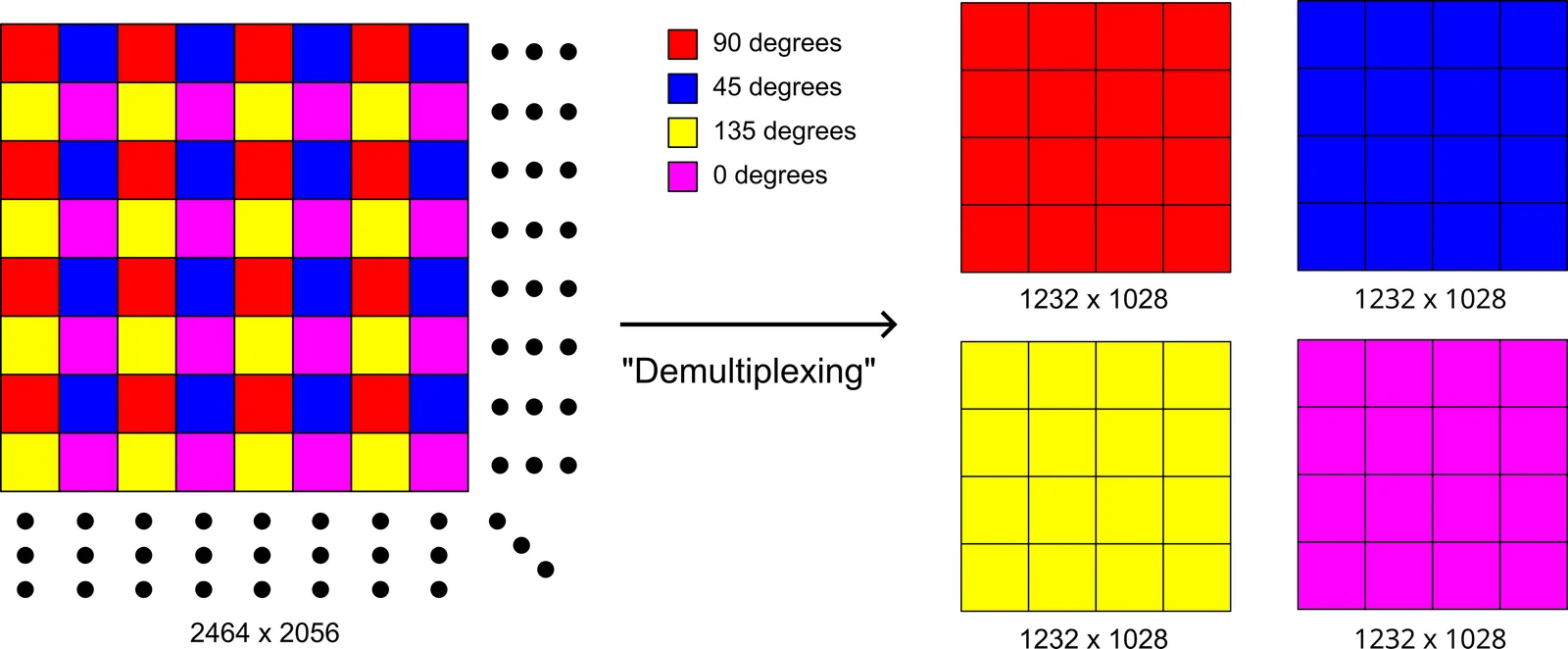
Schematic overview of the Sony Polarsens sensor.

**Figure 3:**
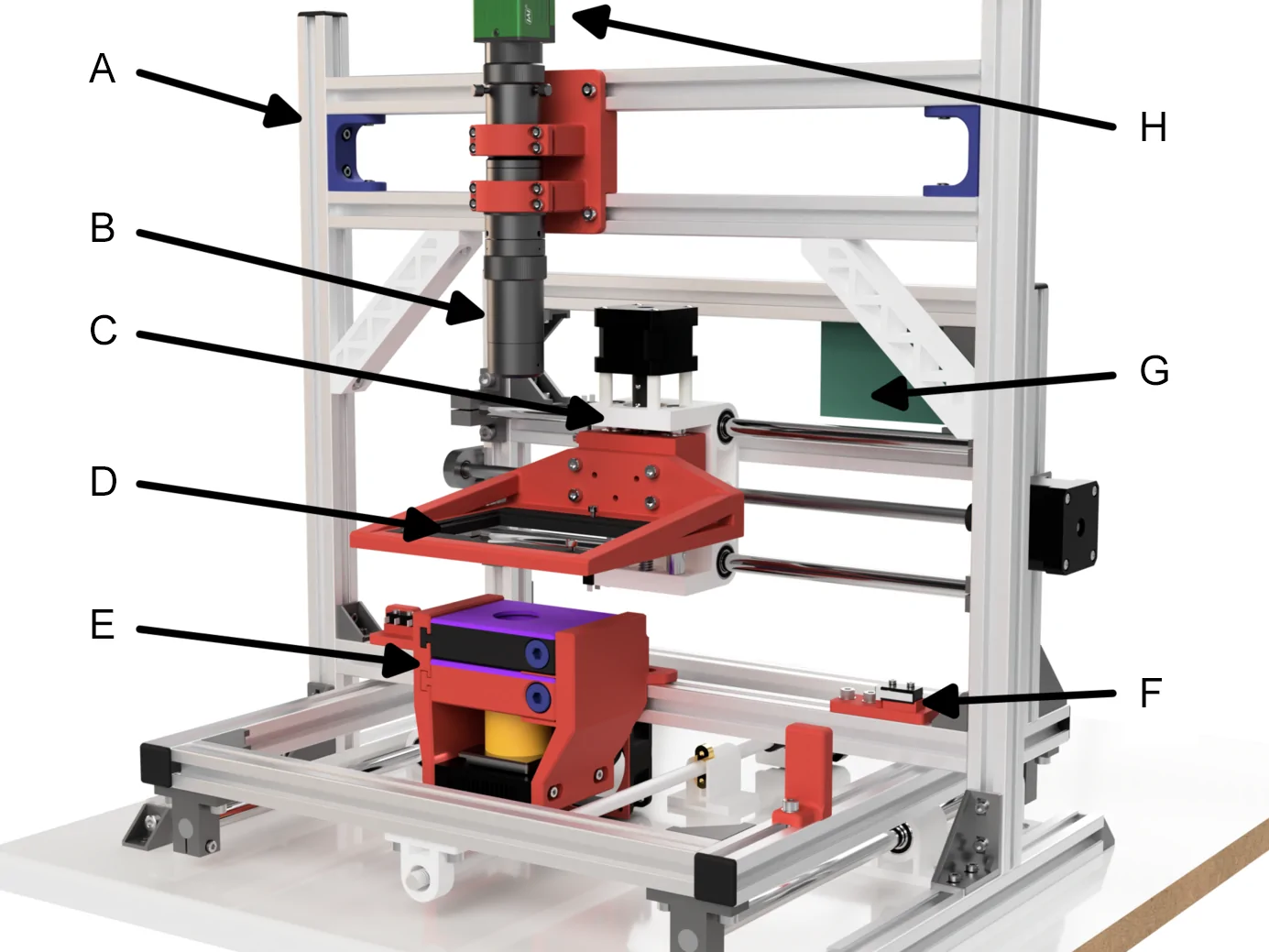
Annotated computer-aided design render of the *Atomicity XtalEyes* plate reader. Components are labelled as follows: A = module gantry, B = lens, C = *x*-axis carriage, D = plate holder, E = light source with filter cassettes, F = limit switches, G = ATMEL-based control board, H = Jai Go-5100 MP.

**Figure 4:**
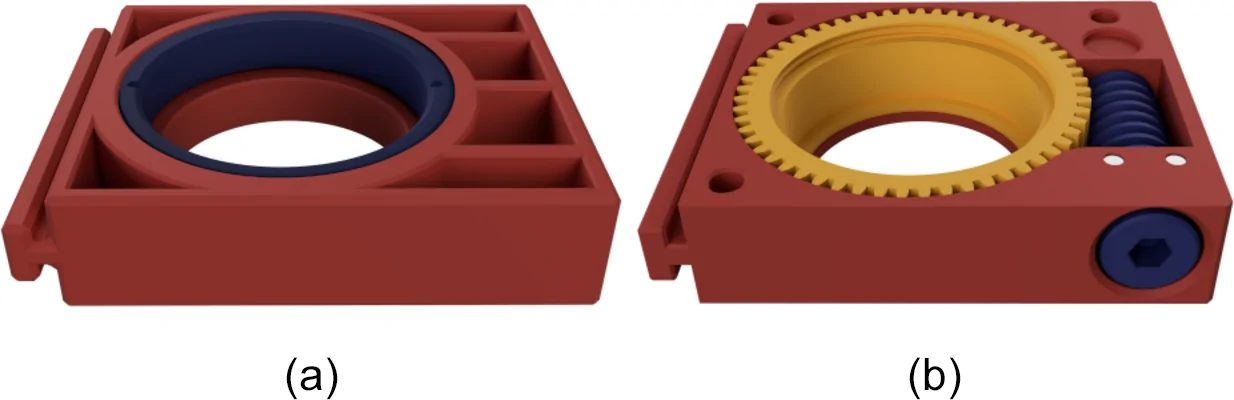
Two primary optical filter cassettes used with the *Atomicity XtalEyes* system. (*a*) A non-rotatable design for rotation-agnostic experiments, and (*b*) a design incorporating rotational alignment.

**Figure 5:**
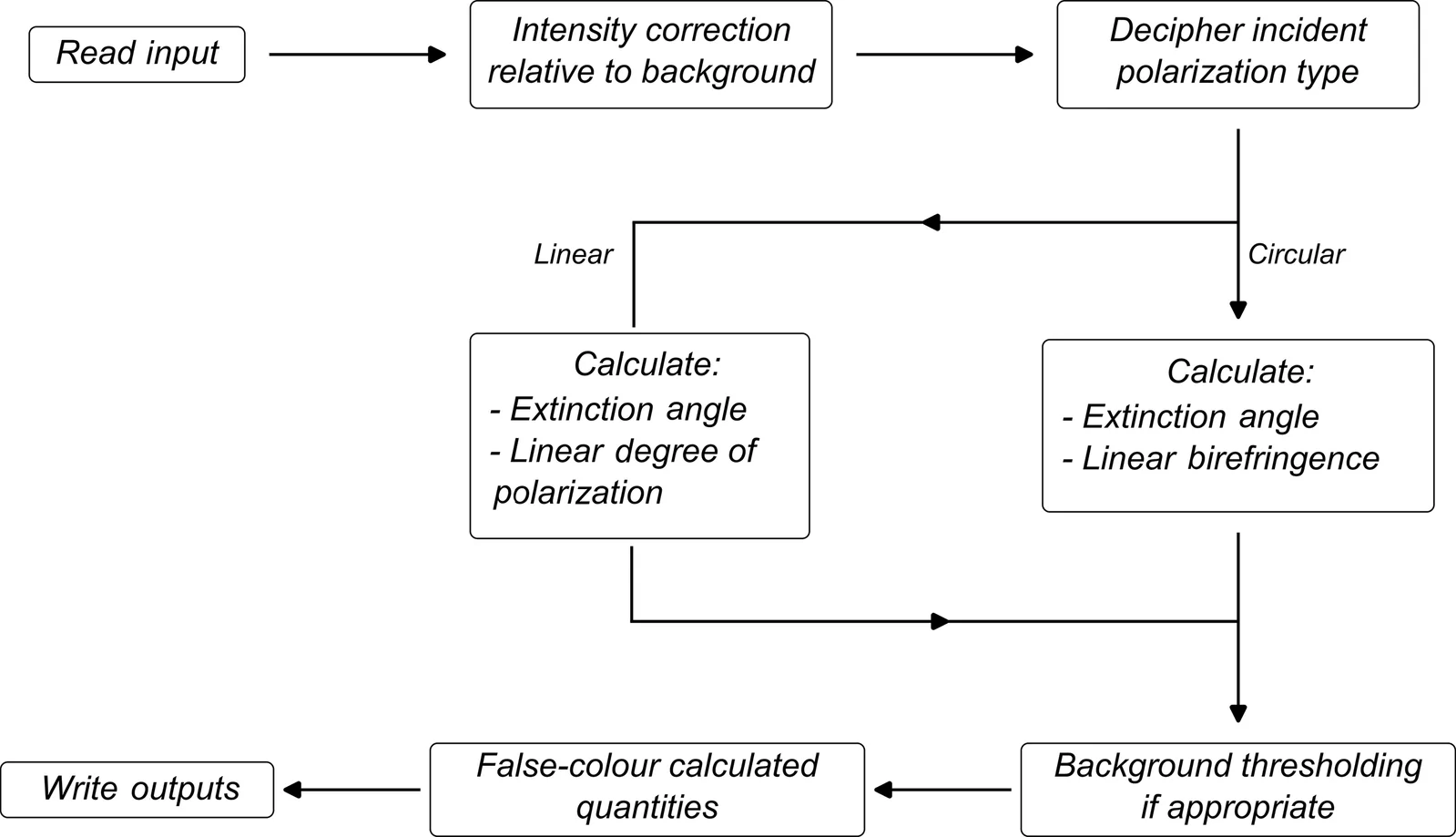
Block diagram representation of the *PolarEyes* software.

**Figure 6:**
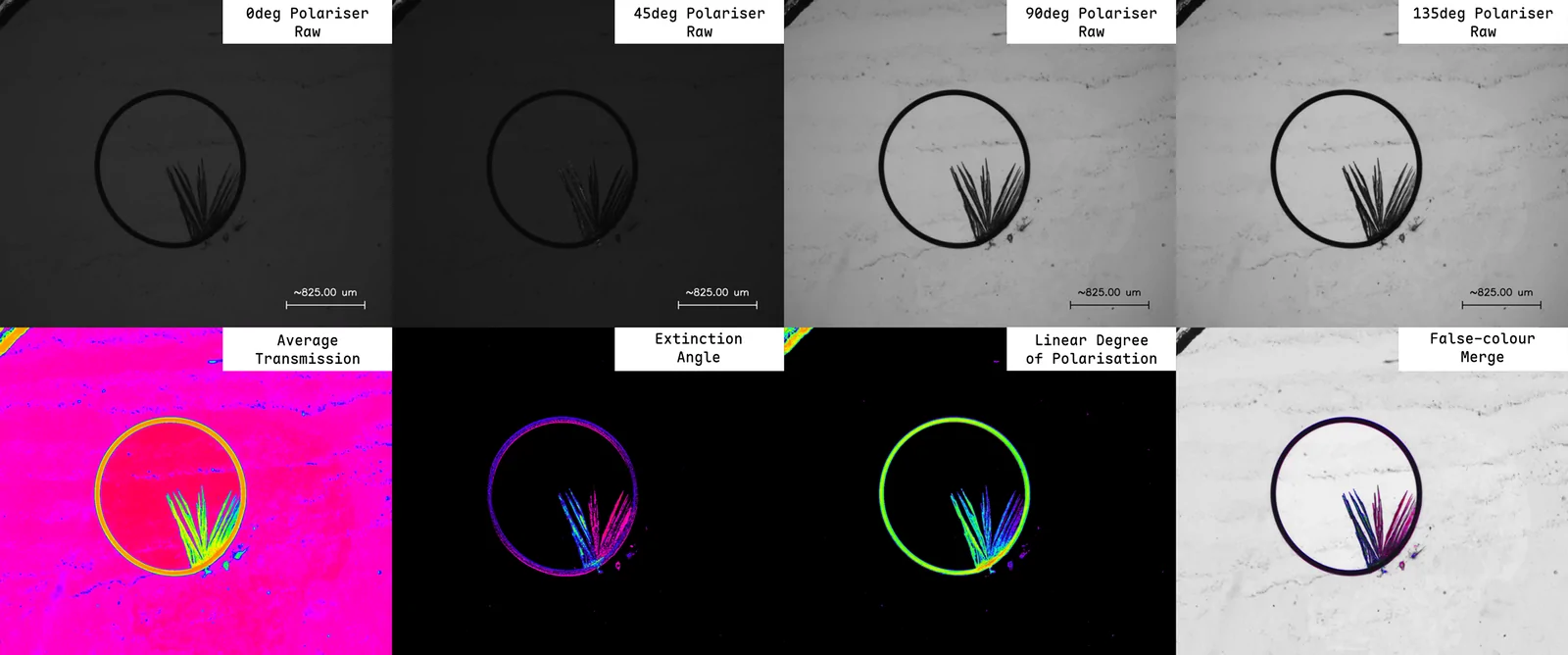
Sample eight-component collage generated after an imaging run.

**Figure 7:**
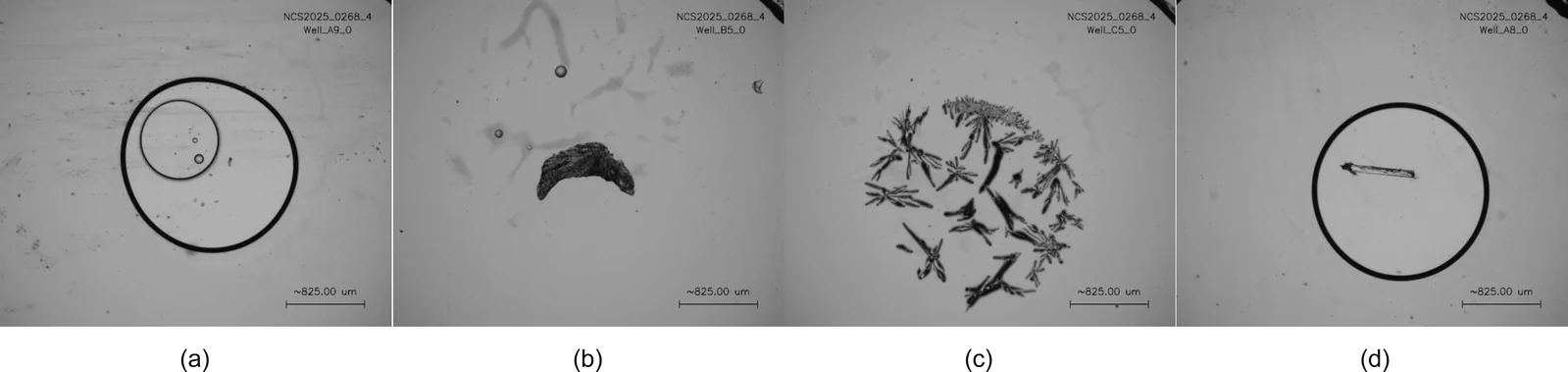
Representative examples of each of the four non-failed ENaCt classes: (*a*) class 1 – still in solution; (*b*) class 2 – deposition of amorphous material; (*c*) class 3 – growth of microcrystalline material not suitable for in-house diffraction equipment; (*d*) class 4 – growth of a single crystal likely to produce structural information when collected in-house.

**Figure 8:**
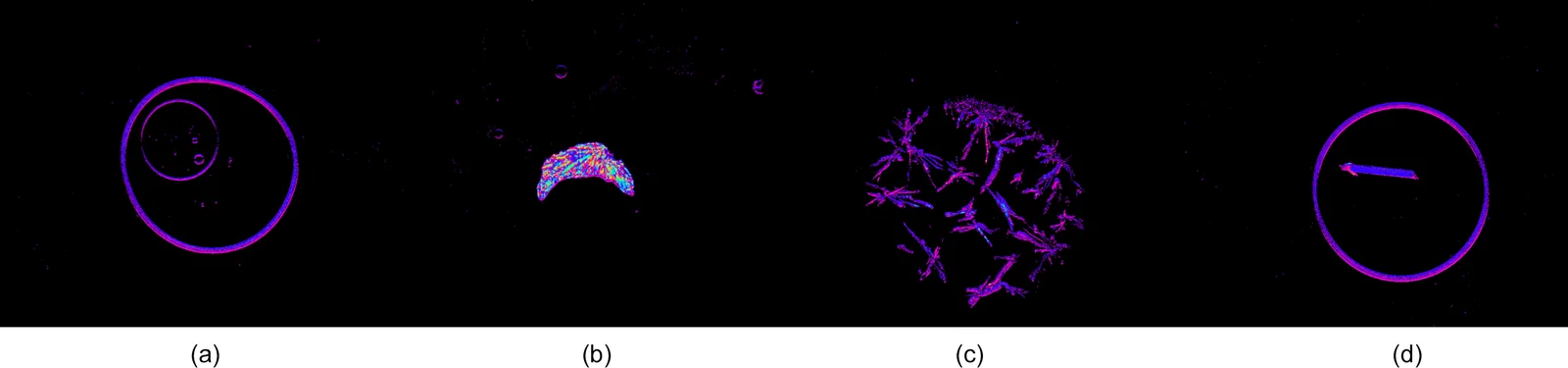
False-coloured polarization angle data for each of the four non-failed ENaCt classes.

**Figure 9:**
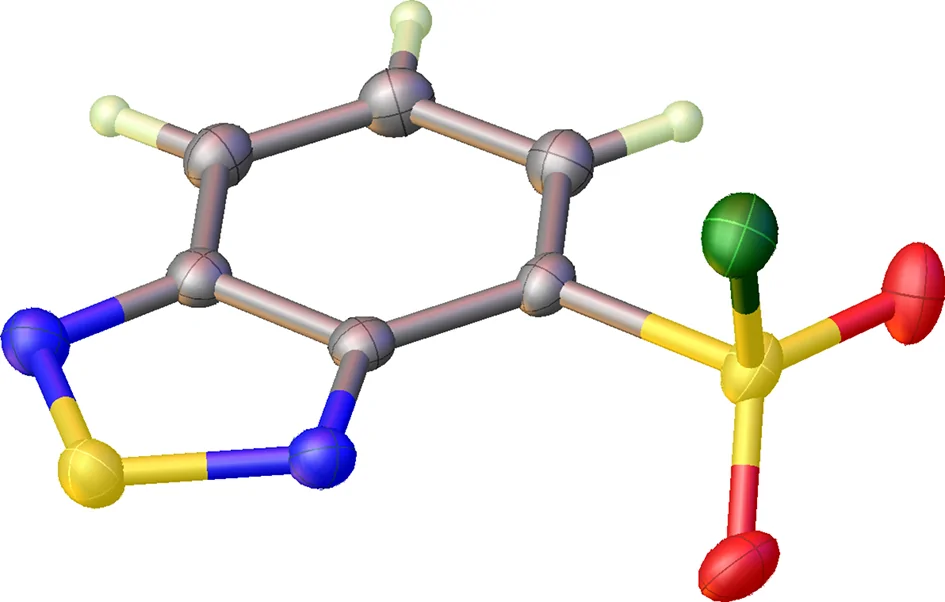
Crystal structure of **A**. Atomic displacement parameters are drawn at the 50% probability level and H atoms are shown as small spheres of arbitrary radii. Key: S yellow, C grey, O red, Cl dark green, H light green and N blue.

**Figure 10:**
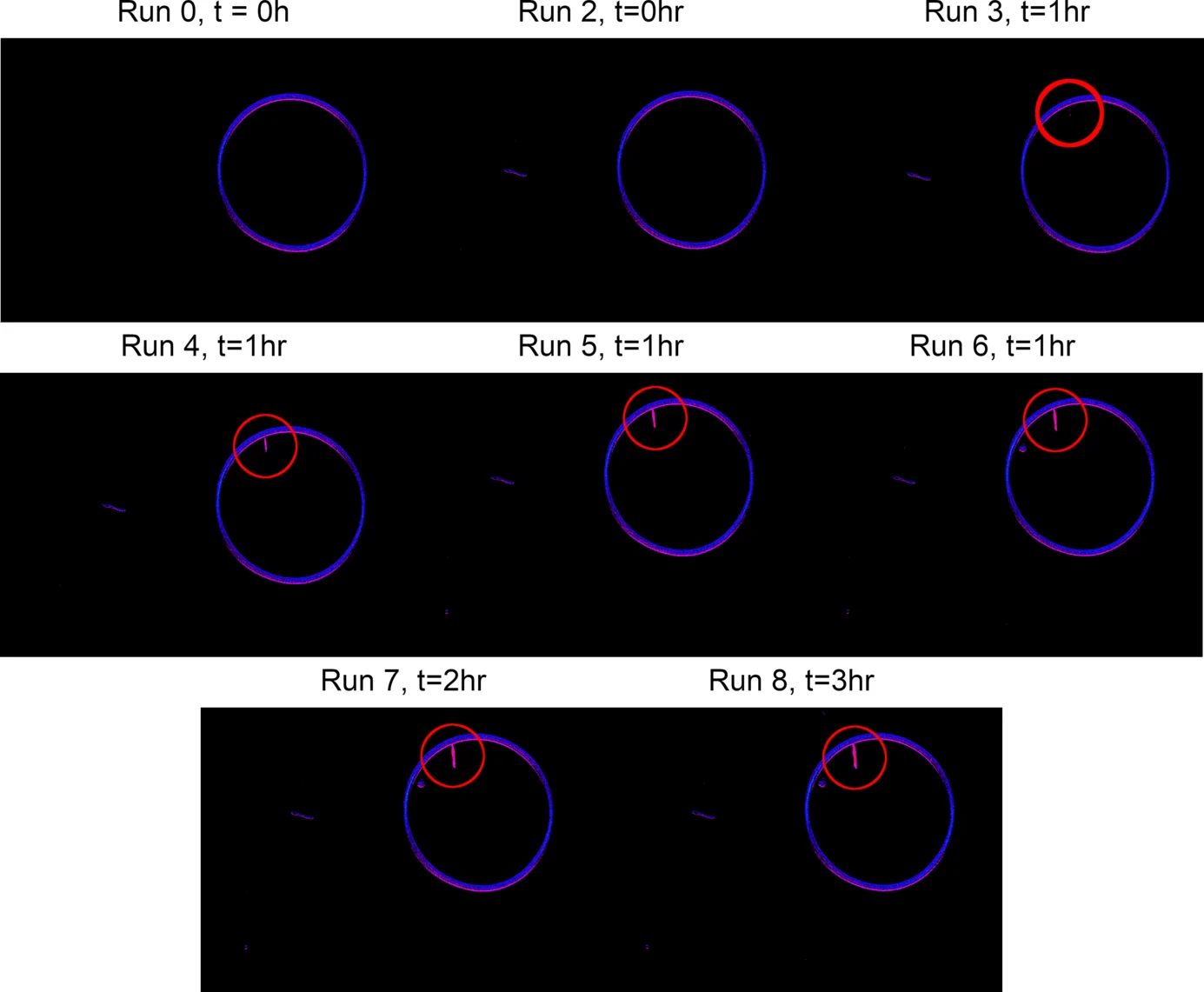
Selected frames from the timelapse study, showing the growth of a crystal of **A** from 1,2-dichloroethane and mineral oil. Extinction angle images generated by *PolarEyes* are shown, with red circles around the growing crystal for clarity.

**Figure 11:**
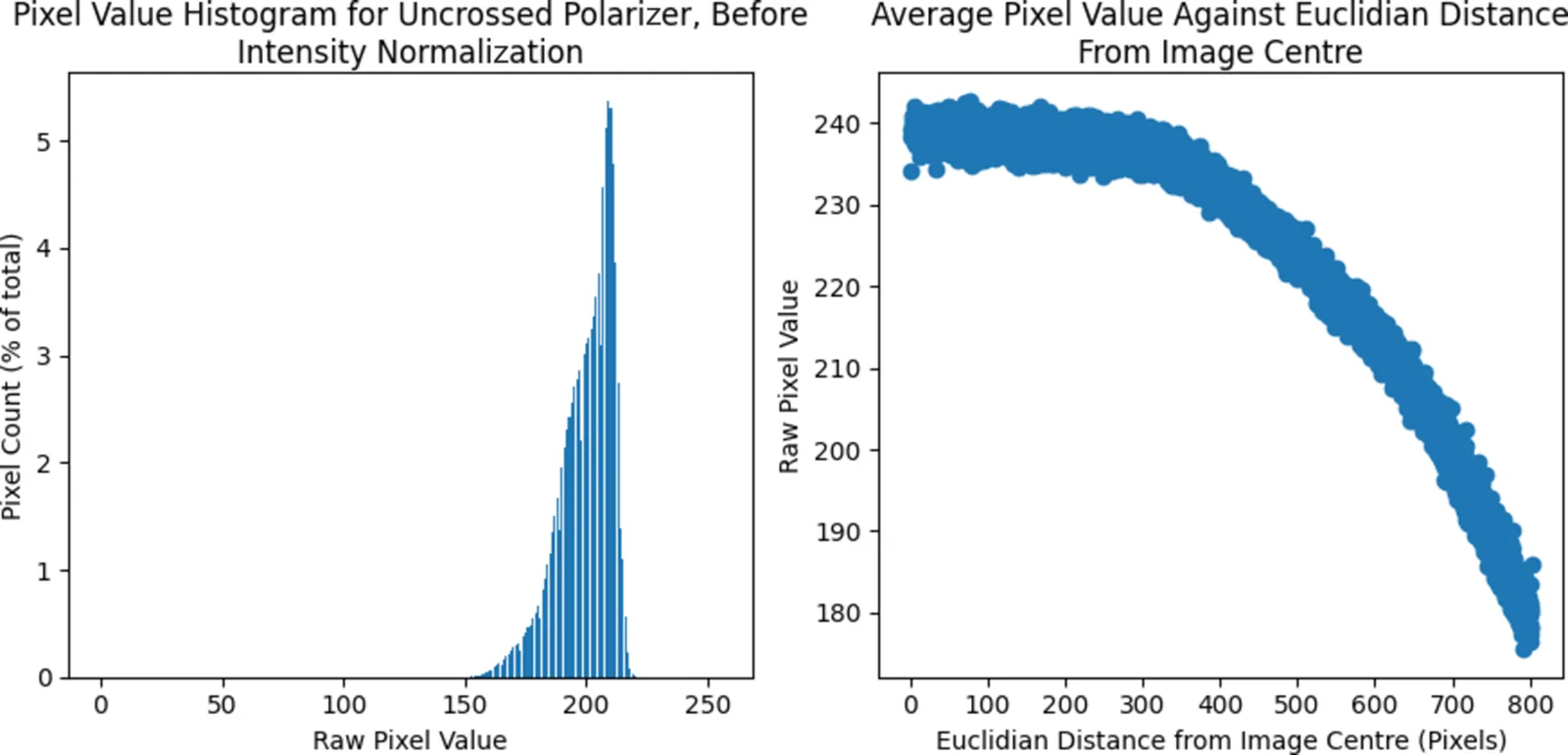
Pixel value plots for 135° polarization angle before normalization.

**Figure 12:**
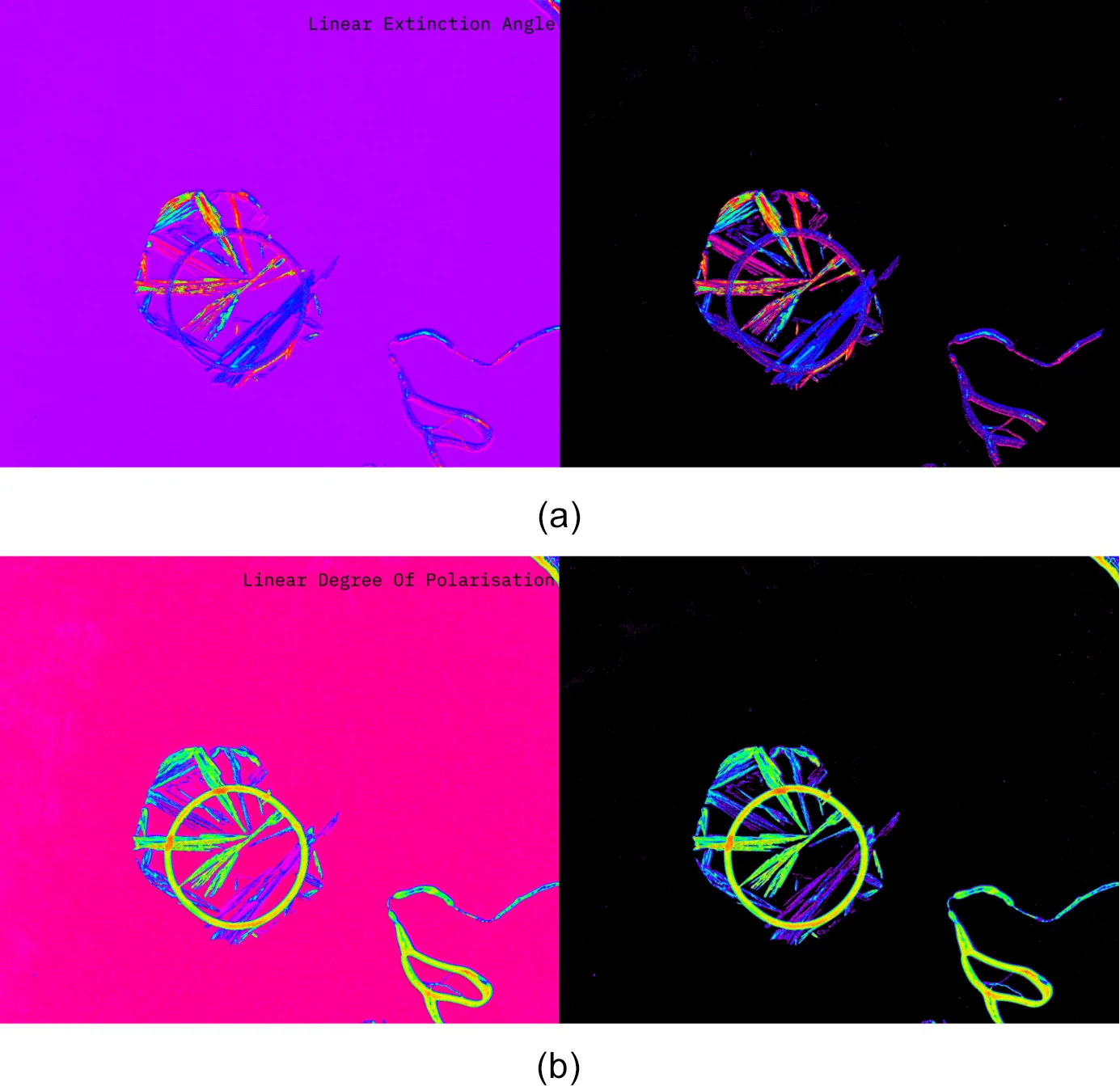
Effect of background correction on *PolarEyes* outputs for (*a*) extinction angle and (*b*) linear degree of polarization.

**Figure 13:**
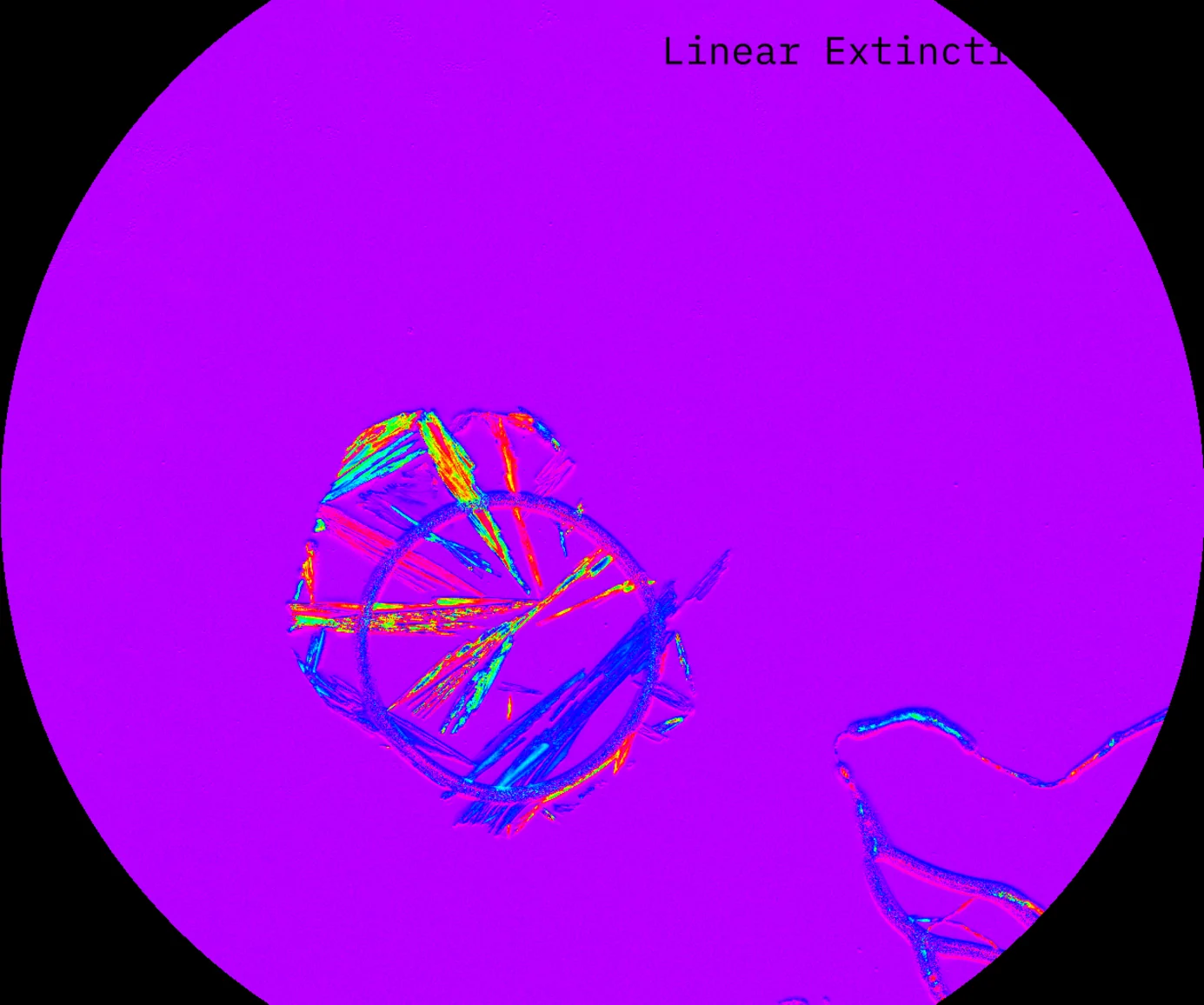
‘Corner clip’ mask visible against an uncorrected extinction angle image.

**Table 1:** Distribution of crystal systems in the major crystallographic databases (CSD, PDB, ICSD) CSD data obtained 2026-07-02, Version 2026.1. PDB data obtained 2026-07-02. ICSD data obtained 2026-02-20.

	Triclinic	Monoclinic	Orthorhombic	Tetragonal	Trigonal	Hexagonal	Cubic
CSD	23.64%	52.76%	20.47%	1.41%	1.16%	0.35%	0.16%
PDB	4.13%	28.04%	35.88%	11.95%	10.46%	7.5%	2.03%
ICSD	4.07%	16.92%	20.87%	15.29%	10.53%	10.96%	21.36%

**Table 2:** The type, volume and concentration of stock solutions used in the ENaCt experiments resulting in polymorph I

Solvent	Volume of solvent added to vials of **A** (µl)	Approximate concentration (mg ml^−1^)
Acetonitrile (MeCN)	150	33.3
Ethyl acetate (EtOAc)	300	16.7
1,2-Dichloroethane (DCE)	400	12.5
Nitromethane (MeNO_2_)	250	20

**Table 3:** Standard plate layout used for the ENaCt experiments Bold numbers **1**–**12** and letters **A**–**H** identify the 96 wells. PMDSO = poly­(dimethyl siloxane) and FC-40 = Fluorinert FC-40.

			Oil (200 nl)											
Plate No.	Solvent (50 nl)		**1**	**2**	**3**	**4**	**5**	**6**	**7**	**8**	**9**	**10**	**11**	**12**
1	Solvent *A*	**A**	No oil	PDMSO					No oil	Fomblin-Y				
		**B**	No oil	FC-40					No oil	Mineral oil				
2	Solvent *B*	**C**	No oil	PDMSO					No oil	Fomblin-Y				
		**D**	No oil	FC-40					No oil	Mineral oil				
3	Solvent *C*	**E**	No oil	PDMSO					No oil	Fomblin-Y				
		**F**	No oil	FC-40					No oil	Mineral oil				
4	Solvent *D*	**G**	No oil	PDMSO					No oil	Fomblin-Y				
		**H**	No oil	FC-40					No oil	Mineral oil				

**Table 4:** Type, volume and concentration of stock solutions used in the ENaCt experiments resulting in polymorph II

Solvent	Volume of solvent added to vials of **A** (µl)	Approximate concentration (mg ml^−1^)
Acetonitrile (MeCN)	150	33.3
Ethyl acetate (EtOAc)	250	16.7
1,2-Dichloroethane (DCE)	300	12.5
Nitromethane (MeNO_2_)	200	20

**Table 5:** Selection of instrument configuration metadata from a sample data file

Key	Value
jai_microscope_camera_pixel_width	3.45
jai_microscope_camera_pixel_height	3.45
jai_microscope_magnification	2.0
jai_microscope_optic_element_0	Linear polarizer; ∼0.15% transmission (crossed polar); 53999 by Edmund Optics
jai_microscope_optic_element_1	Zoom lens; 2× mag.; mags = 0.75,1,1.5,2,3; WD = 56 mm; VZM 300i by Edmund Optics

## Data Availability

Copies of the *PolarEyes* source code are available from the *Atomicity* Gitlab repository at https://gitlab.com/atomicity-2/polareyes. Crystallographic data have been deposited in the Cambridge Structural Database.

## Appendix A Experimental

The crystallization of benzo-2,1,3-thiadiazole-4-sulfonyl chloride, **A**, was carried out using ENaCt protocols. **A** (5 mg in total) was weighed and transferred to four 1.75 ml screw-top vials. Four different organic solvents (Table 2 ▸) were subsequently added in 50 µl portions to each individual vial until the sample was fully dissolved, up to a maximum of 400 µl of solvent.

The stock solutions of **A** (50 nl) were dispensed via an SPT Labtech Mosquito Xtal3 liquid-handling robot into 96-well glass plates (SWISSCI LCP Modular, 1 mm glass base with 100 µm adhesive spacer) containing either an appropriate crystallization oil (200 nl) or no oil (Table 3 ▸). For wells containing oil, the oil was dispensed prior to injection of the stock solution into the oil droplet. Plates were sealed with a glass cover slip (SWISSCI LCP Modular, 175 µm glass) and allowed to stand undisturbed at room temperature. A single crystal of **A** grown from MeCN (50 nl, 33.3 mg ml^−1^) encased in a 200 nl droplet of Fomblin-Y oil (Plate 1, well **A8**) was subsequently analysed by SCXRD after two weeks.

Wells were opened using a tungsten carbide scriber to remove a small portion of the glass cover slide, and the crystal was manipulated using Hampton Research microtools. Crystals were transferred to a glass slide, extracted under oil (Fomblin-YR 1800) and mounted onto a 35 µm MiTeGen Kapton loop before being flash-cooled to 150 K under N_2_ using an Oxford Cryosystems Cryostream cooler prior to collection. SCXRD data for **A** were collected on a Rigaku XtaLAB Synergy-S diffractometer, fitted with a PhotonJet micro-focus sealed X-ray tube (Cu *K*α radiation, λ = 1.54184 Å, four-circle goniometer, HyPix-Arc 100° detector). Unit-cell measurement, data collection and data reduction were performed using the Rigaku software *CrysAlisPRO*. A numerical absorption correction was applied therein using Gaussian integration over a multi-faceted crystal model. The structure of **A** was solved using *SHELXT* (Sheldrick, 2015 ▸) and refined using *SHELXL* (Sheldrick, 2008 ▸) through the *Olex2* (Dolomanov *et al.*, 2009 ▸) interface. All non-hydrogen atoms were refined using anisotropic displacement parameters. All hydrogen atoms were placed geometrically and refined using a riding model. Crystallographic data and specific refinement details can be found within the CIF in the supporting information, and are also deposited with the Cambridge Crystallographic Data Centre (deposition num­bers 2469226 for polymorph I and 2536919 for polymorph II).

A crystal of polymorph II was grown, harvested, collected and refined using the same methodology and conditions, except that the solution concentrations were slightly increased. The new concentrations are shown in Table 4 ▸.

## Appendix B *PolarEyes* detail

Several details regarding the internal operation of the *PolarEyes* application were omitted from the main text for the sake of clarity. For the interested reader, some lower-level details are presented here.

### b1. Normalization

Mentioned previously were the attempts made at physically ensuring homogeneity in the light source, but there is a point at which the cost of additional optical components and engineering effort surpasses the benefits. To try and eliminate the last significant inhomogeneity, the background image is used to derive a multiplicative mask based on the deviation from the maximum value for each polarization angle. Each mask is then applied to its corresponding polarization angle in the input data before any processing is performed.

Note that polarization quantities are also calculated for the intensity-corrected background image, producing a series of images against which the quantities calculated for each well may be compared.

The case for performing additional software-based normalization is made by the graphs shown in Fig. 11 ▸. Shown in the left-hand panel is a count of each pixel value present in the unnormalized 135° background image. An ideal image with no inhomogeneity and perfect exposure would have a single peak at the far right-hand side, indicating that every pixel was almost fully saturated and had a very similar value. As it stands, we have a reasonably broad range of pixel values leading up to a slightly underexposed peak. Shown in the right-hand panel is a plot of average pixel value against Euclidian distance from the image’s centre. From this it is easily seen that the intensity is reasonably constant for a central circle around 600 pixels in diameter, outside of which we begin to lose intensity, with the lowest intensity out in the far corners. However, even at the lowest value pixels are still at ∼80% of the maximum possible intensity and ∼75% of the maximum observed intensity. Since in our setup the camera is configured to use an 8-bit analogue-to-digital converter to digitize the raw light intensity, the maximum possible value is 255 (higher bit depths are available but we elected to avoid them to avoid extending the data storage requirements of the system yet further). This is not unexpected – vignetting is a well understood issue in microscopy – and in the future we may seek to address this issue more formally than our current naïve implementation.

It would be possible to embed each background image statically in the instrument and have corrections made automatically. However, we chose to take a background image at the beginning of every run and have *PolarEyes* calculate a new background every time, to take better account of environmental factors such as LED output degradation.

Despite the sub-optimal initial flatness, the light source (and by extension, the instrument) is producing very valuable data as is, showing that even imperfect setups like this can still be valuable tools. In particular, the 600 pixel circle of even illumination represents a real-space area of ∼1 mm^2^ with our usual 2× magnification; this is the area in which most droplets should be dispensed, and thus most crystallizations *should* occur.

### b2. Background correction

*PolarEyes* supports two background correction regimes: skipping the background correction entirely if, for example, the background contains useful information; or a pixel-wise comparison operation between foreground and background and a provided threshold. If the difference exceeds the threshold then the pixel value is unchanged; otherwise it is set to zero and will be ignored during a false-colouring operation. Each calculated quantity (polarization angle, degree of polarization *etc.*) may be handled differently in both method and threshold.

Fig. 12 ▸ shows the extinction angle and degree of polarization both before and after background correction. The extinction angle image [Fig. 12 ▸(*a*)] shows a purple background in the majority of the image, corresponding to large areas in which crystalline material has not grown and the un-rotated light passes straight to the sensor.

It is evident how this correction routine vastly improves the legibility of the output image by reducing the visual clutter, allowing the user to focus easily on an area of interest. Future versions of *PolarEyes* may include more sophisticated background-correction algorithm(s), such as automatically deriving the threshold based on either global or local image statistics.

A further measure taken to prevent visual noise is a circular mask applied to images. Small misalignments of either the plate in the holder or the adhesive spacer on the plate, or inaccuracies in the instrument’s translation, can cause the spacer to appear in the captured images. As with many adhesives, the spacer is both wildly and inconsistently polarizing, which can cause large blocks of colour to creep in from the corners. This can be seen in the greyscale images in Fig. 6 ▸, wherein the spacer appears in both upper corners of each image. As a result of the masking operation, however, that visual noise is absent from the extinction angle image.

The mask is easily seen if the background correction is disabled (as shown in Fig. 13 ▸). A circle with a diameter equal to the image width is used, and any pixel outside of that circle has its value set to black. A downside of this brute-force approach is also shown in Fig. 13 ▸: the system is not intelligent about applying corrections. It is possible for real data to be masked out through this clipping procedure, although in practice we have found that the material we are interested in grows in the centre of the well and so this does not pose a huge problem. Future versions of *PolarEyes* could solve this problem by applying more advanced computer vision techniques to detect when objects from within the ‘allowed region’ cross the border, and then not performing the clipping on that corner or at all.

### b3. Data input

It was briefly covered in Section 4 that the imaging system stores data using the hierarchical data format (HDF). Aspects of the instrument’s configuration are stored as metadata for the global file object, and this includes the polarizing optics placed in the light path before the sample. Examples of these metadata are shown in Table 5 ▸.

The keys present at the global and dataset levels are *de facto* standardized across the system, and this enables different software components to interact with the imaging instrument’s outputs. *PolarEyes* uses this stored information to decipher the polarization state incident on the sample and changes its internal behaviour accordingly to use either the linear- or the circular-specific algorithm.

Checks are also performed to ensure that the processing performed is sensible: the camera sensor must be one of the few models with an on-chip polarizer fitted; the pixel size and total magnification must be known in order to calculate surface area and discard uninteresting contours; and finally the plate identifier and well address must be known for output file path generation.

Some of this information is carried over into any produced output files where metadata are supported, and at the moment this is limited only to the HDF5 output mode.
